# Integrative analysis of HASMCs gene expression profile revealed the role of thrombin in the pathogenesis of atherosclerosis

**DOI:** 10.1186/s12872-023-03211-0

**Published:** 2023-04-12

**Authors:** Yichen Zhang, Lin Sun, Xingsheng Wang, Qingbo Zhou

**Affiliations:** 1 The Second Hospital of Shandong University, Jinan, Shandong Province China; 2 Jinan Central Hospital, Shandong University, Jinan, Shandong Province China

**Keywords:** Thrombin, Smooth muscle cells, Atherosclerosis, Inflammatory, Proliferation, Foam cell

## Abstract

We explored the effect of thrombin on human aortic smooth muscle cells (HASMCs) and further analyzed its role in the pathogenesis of atherosclerosis (AS). Thrombin-induced differentially expressed genes (DEGs) in HASMCs were identified by analyzing expression profiles from the GEO. Subsequently, enrichment analysis, GSEA, PPI network, and gene-microRNAs networks were interrogated to identify hub genes and associated pathways. Enrichment analysis results indicated that thrombin causes HASMCs to secrete various pro-inflammatory cytokines and chemokines, exacerbating local inflammatory response in AS. Moreover, we identified 9 HUB genes in the PPI network, which are closely related to the inflammatory response and the promotion of the cell cycle. Additionally, we found that thrombin inhibits lipid metabolism and autophagy of HASMCs, potentially contributing to smooth muscle-derived foam cell formation. Our study deepens a mechanistic understanding of the effect of thrombin on HASMCs and provides new insight into treating AS.

## Introduction

Thrombin, a serine protease, is one of the key players in the coagulation cascade. In addition to its pro-coagulation and anticoagulant activities, thrombin plays various physiological roles through protease-activated receptors (PARs), including vasomotor regulation. Also, thrombin establishes a strong link between coagulation and inflammation [1]. Besides platelets, PARs are also expressed in vivo by the endothelium of normal-appearing human arteries. Furthermore, PARs are widely expressed in atherosclerotic arteries, including areas rich in smooth muscle cells(SMCs) [2]. Active thrombin was detected in the neointima of human atherosclerotic arteries, and its concentrations were determined to correspond to a range of concentrations necessary for optimal PAR-1 activation [3].

Atherosclerosis (AS) is a chronic inflammatory disease of the vessel wall primarily driven by an innate immune response through myeloid cells like monocytes and macrophages [4]. The smooth muscle cell (SMC) plays a crucial role in atherosclerosis development [5]. According to previous studies, thrombin mainly promotes SMC proliferation and the expression of cytokines and chemokines in SMCs [6]. Pro-inflammatory cytokines promote pathological production of thrombin, and thrombin aggravates the inflammatory response by activating PARs. The two amplify each other’s effects, leading to the development and progression of AS [7]. New studies report that thrombin can promote the formation of smooth muscle-derived foam cells [8, 9]. These findings suggest that the effects of thrombin on SMC are complex and varied, and the role of thrombin in the pathogenesis of AS still needs to be further explored.

In this study, we confirmed that thrombin promotes inflammatory response and cell cycle progression in HASMCs. Additionally, we found that thrombin inhibits lipid metabolism and autophagy of HASMCs, potentially contributing to smooth muscle-derived foam cell formation. Our study provides many new insights into the role of thrombin in AS pathogenesis.

## Material and methods

### Analysis scheme

The present study was designed to identify thrombin-induced differentially expressed genes (DEGs) in human aortic smooth muscle cells (HASMCs) through analysis of mRNA expression profiles downloaded from the Gene Expression Omnibus (GEO) database. We used Gene Ontology (GO), Kyoto Encyclopedia of Genes and Genomes (KEGG), and Gene set Enrichment Analysis (GSEA) to study the molecular mechanism of thrombin’s effect on HASMCs. Using String [10], we constructed a protein–protein interaction (PPI) network and identified hub genes using Cytohubba [11] and MCODE [12], two Cytoscape [13] plug-ins. Utilizing the miRWalk website, we analyzed miRNAs that regulate target genes.

### Microarray data collection and quality control

The mRNA microarray data were obtained from the GEO database (http://www.ncbi.nlm.nih.gov/geo) with accession number GSE104499. An Illumina HumanWG-6 v3.0 expression beadchip platform was used. Quiescent HASMCs were treated with and without thrombin (0.5 U/ml) for 8 h, and total cellular RNA was extracted by Trizol reagent. A total of three normal control samples and three thrombin intervention samples were obtained. We studied intra-group and intergroup differences using principal component analysis (PCA) and correlation matrix. The ggplot2 [14],corrplot [15], and factoextra [16] packages of the R programming language are used for data visualization.

### Identification of differentially expressed genes

After standardizing the datasets by quantiles, We used the limma V3.46.0 (linear models for microarray data) package of the R software program (version 4.0.3) to screen DEGs [17]. Our selection criteria considered only genes with a log2 fold change (FC) > 2 and FDR < 0.01. Visualizing the DEGs was achieved by using volcano plot filtering. Each dataset’s heatmaps of DEGs were created using the R software package Pheatmap V1.0.12 [18].

### Enrichment analysis of functions and pathways

GO enrichment analyses were performed with Metascape [19], and terms with *P* < 0.01 were considered significant. Thirty-one terms were enriched for up-regulated genes, while thirteen were enriched for down-regulated genes. The KEGG pathway enrichment analysis of DEGs was automated and visualized by ClueGo [20] and Cluepedia [21] plug-ins in Cytoscape software (version 3.8.2) with a kappa score ≥ 0.4. Only pathways with *P* < 0.05 were considered meaningful. Using ClusterProfiler V3.18 [22], an R-dependent Bioconductor software package, GSEA was performed on all genes that had been detected. Gene sets were considered to be significantly enriched with *P* < 0.01. According to GSEA results, 291 GO terms and 47 KEGG pathways were activated, whereas 69 GO terms and 12 KEGG pathways were inhibited. Data visualization is performed by the enrichplot [23] package of the R programming language.

### Construction of PPI network and the miRNA regulatory network

We built a network of PPI using String, after which hub genes were identified using Cytohubba and MCODE. Cytohubba determined the top 10 genes based on node degree in the network. A cluster of 10 nodes and 36 edges is found in the network by MCODE(MCODE score ≥ 8). The intersection of those two results was determined to be the hub genes. Using the network tool miRWalk [24], we established the miRNA regulatory network between thrombin(F2) and the target genes. Cytoscape was used to visualize the networks.

### Reagents and chemicals

ox-LDL(YB-002) was purchased from Yiyuan Biotechnologies (Guangzhou, China); Thrombin (T6884), dimethyl sulfoxide (DMSO; D5879) were purchased from Sigma-Aldrich (St. Louis, MO, USA); Fetal bovine serum (FBS; 10,099–141) from Gibco; PrimeScript™ RT reagent Kit and TB Green® Premix Ex Taq from Takara (Dalian, China). Oil Red O (G1262) was purchased from Solarbio (Beijing, China).

### Cell lines, cell culture and drug treatment

Rat aorta vascular smooth muscle cells (RA-VSMCs) were purchased from the American Type Culture Collection (ATCC, Manassas, VA, USA). Cells were cultured in in Dulbecco’s modified Eagle’s medium (DMEM glucose 5.5 mmol/l) supplemented with 10% fetal bovine serum (FBS, Gibco), penicillinG (100 U/mL), and streptomycin (100/mL) in a 5% CO2 incubator at 37° C. Cells of the 4–8 generation were employed in all experiments. In experiments for thrombin stimulation, cells at 60–70% confluence were treated with various doses of thrombin (1, 2, or 4U/ml) for 2 h.

### RNA extraction and quantitative real-time polymerase chain reaction

The total RNA was extracted from RA-VSMCs using Trizol RNA-RNAiso Plus. Reverse transcription was conducted to generate complementary DNA (cDNA) using the Prime Script RT reagent kit with gDNA Eraser according to the manufacturer’s protocol. The mRNA expression of IL-6, and ABCA1 was determined by quantitative real-time PCR using SYBR premix Ex Tap TM (TLiRNSEHPLUS). Predesigned primers were as follows: β-actin: 5’-CTCTGTGTGGATTGGTGGCT-3’ (forward primer), 5’-CGCAGCTCAGTAACAGTCCG-3’ (reverse primer); ABCA1: 5’-GCAGCGACCATGAAAGTGAC-3’ (forward primer), 5’-GAGGCGGTCATCAATCTCGT-3’ (reverse primer);IL6:5’-TTTCTCTCCGCAAGAGACTTCC-3’ (forward primer), 5’-TGTGGGTGGTATCCTCTGTGA-3’ (reverse primer). β-Actin was used as the internal control. Finally, relative mRNA expressions of these genes were calculated using the 2(− ΔΔCt) method.

### Oil red O staining

The RA-VSMCs were seeded into 6-well plates at 1 × 10^5^ cells per well. The serum-starved cells were treated with 50 μg/ml ox-LDL for 24 h in the presence or absence of thrombin (2U/ml). After being washed with PBS, RA-VSMCs were fixed with 4% paraformaldehyde for 30 min, stained with 0il red O working solutions, and then counterstained with hematoxylin. The positive staining (red) foam cells were photographed under a microscope at 40 × magnification.

## Results

**Fig. 1:**
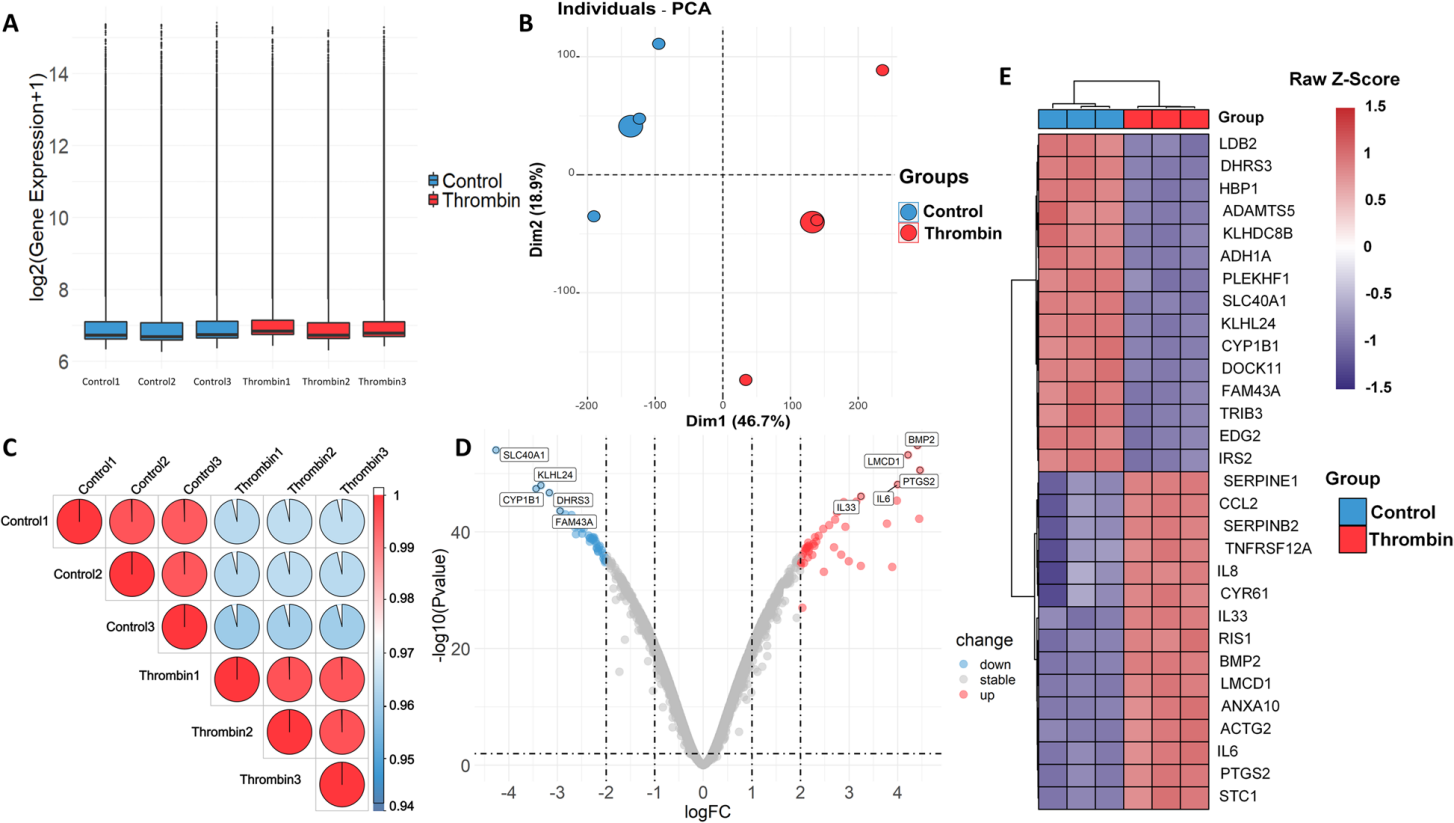
**A** Box plot of normalized gene expression data. Samples are labeled on the x-axis, and gene expression values are labeled on the y-axis. **B** The expression level for all genes in each sample has been reduced to 2 principal components. **C** Correlation matrix between samples. **D** Volcano plot of differentially expressed genes. Label 10 genes that are characterized by the smallest *P* values. **E** Heatmap of top 30 differentially expressed genes identified by *P*-value

After standardizing the data sets (Fig. 1A), we used PCA to reduce the dimension of gene expression levels in each sample and performed correlation matrix analysis on the samples (Fig. 1B and C). The results showed a significant difference between the control and thrombin treatment groups but no significant difference within each group. We identified 91 DEGs in HASMCs that changed their expression more than fourfold in response to thrombin with *P* < 0.01, including 49 up-regulated and 42 down-regulated genes. The DEGs are shown in a volcano plot (Fig. 1D), and the top 30 DEGs, as determined by *P*-value, are shown in a heatmap whose clustering is based on Euclidean distance (Fig. 1E).

### Thrombin promotes the proinflammatory phenotype in HASMCs

**Fig. 2:**
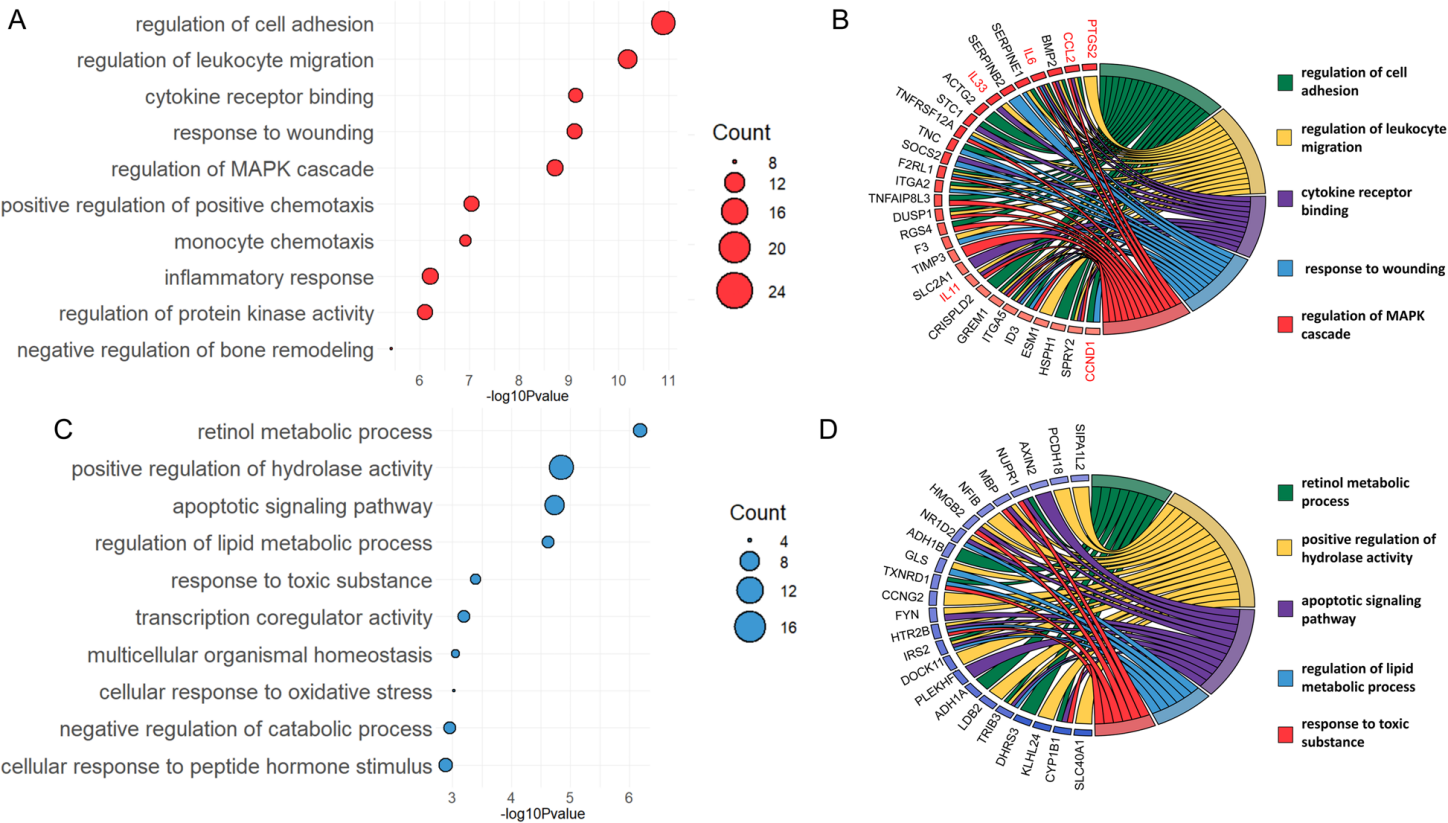
Gene Ontology (GO) enrichment analysis of differentially expressed genes (DEGs). Bubble chart shows the top 5 GO terms enriched by up-regulated (**A**) or down-regulated (**C**) DEGs according to the *P*-value, with bubble size representing the number of genes involved in the terms. Chord plot shows the distribution of DEGs in the top 5 GO terms enriched by up-regulated (**B**) or down-regulated (**D**) DEGs. Symbols of DEG are presented on the left side of the graph in order of fold change. Colored connecting lines determine gene involvement in the GO terms

**Fig. 3:**
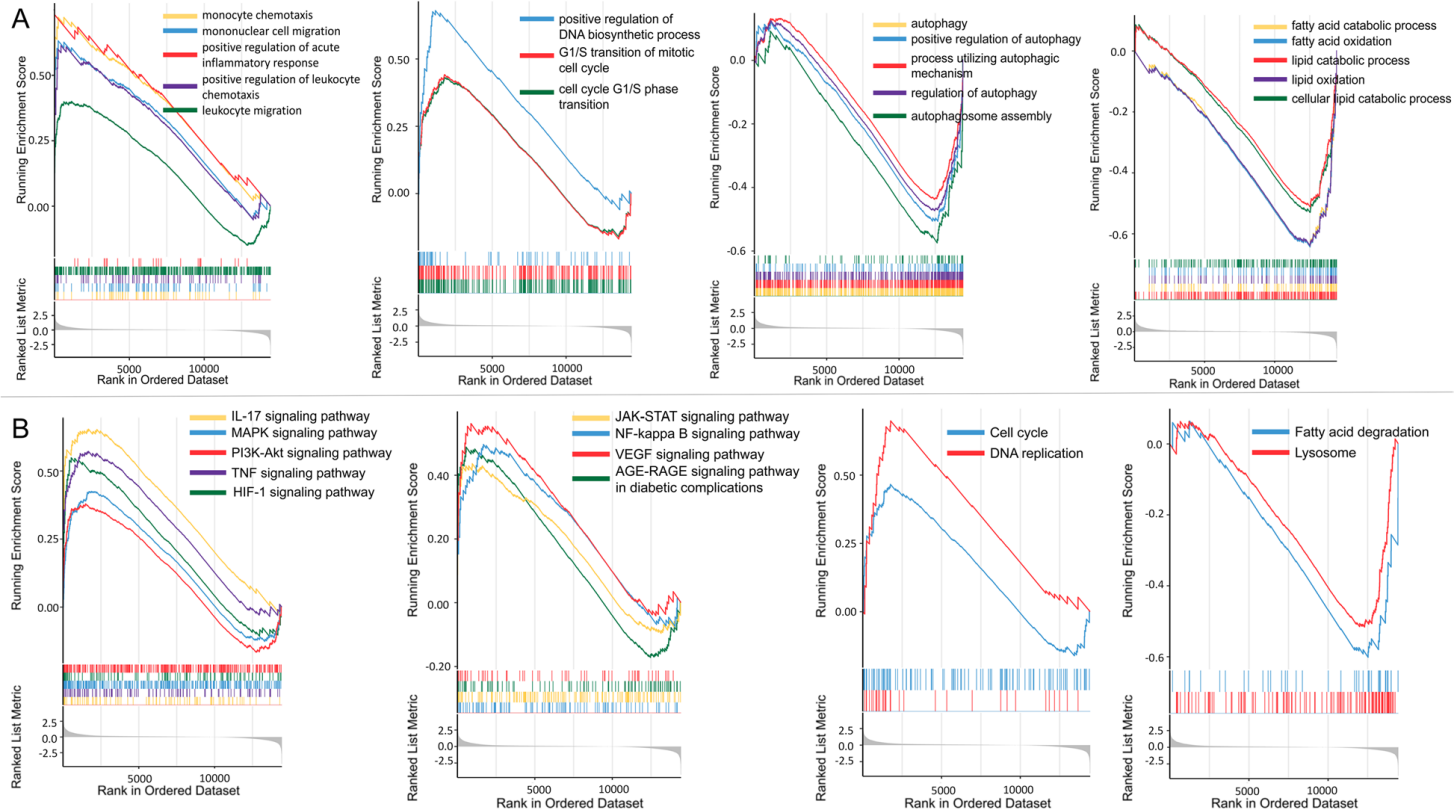
GSEA was performed to investigate thrombin’s potential effect on HASMCs.After eliminating the gene sets associated with the hemostasis and coagulation process as well as RNA and ribosomal biological processes, the representative GO terms (**A**) and KEGG pathways (**B**) are shown in the GSEA plots

**Fig. 4:**
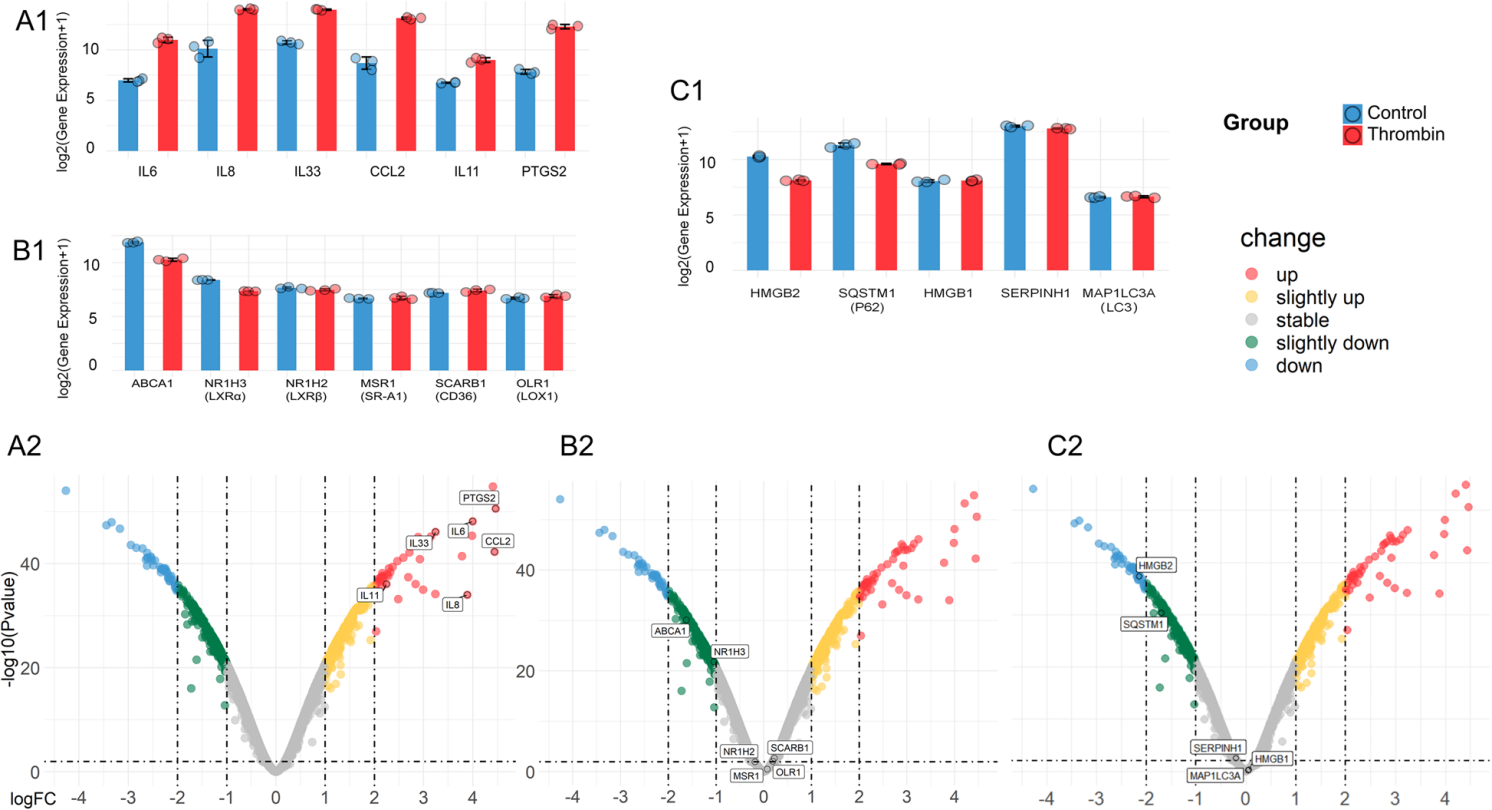
Bar graphs and Volcano plots of certain normalized gene expression data associated with inflammation (**A**1&2), lipid metabolism (**B**1&2), and apoptosis (**C**1&2)

**Fig. 5:**
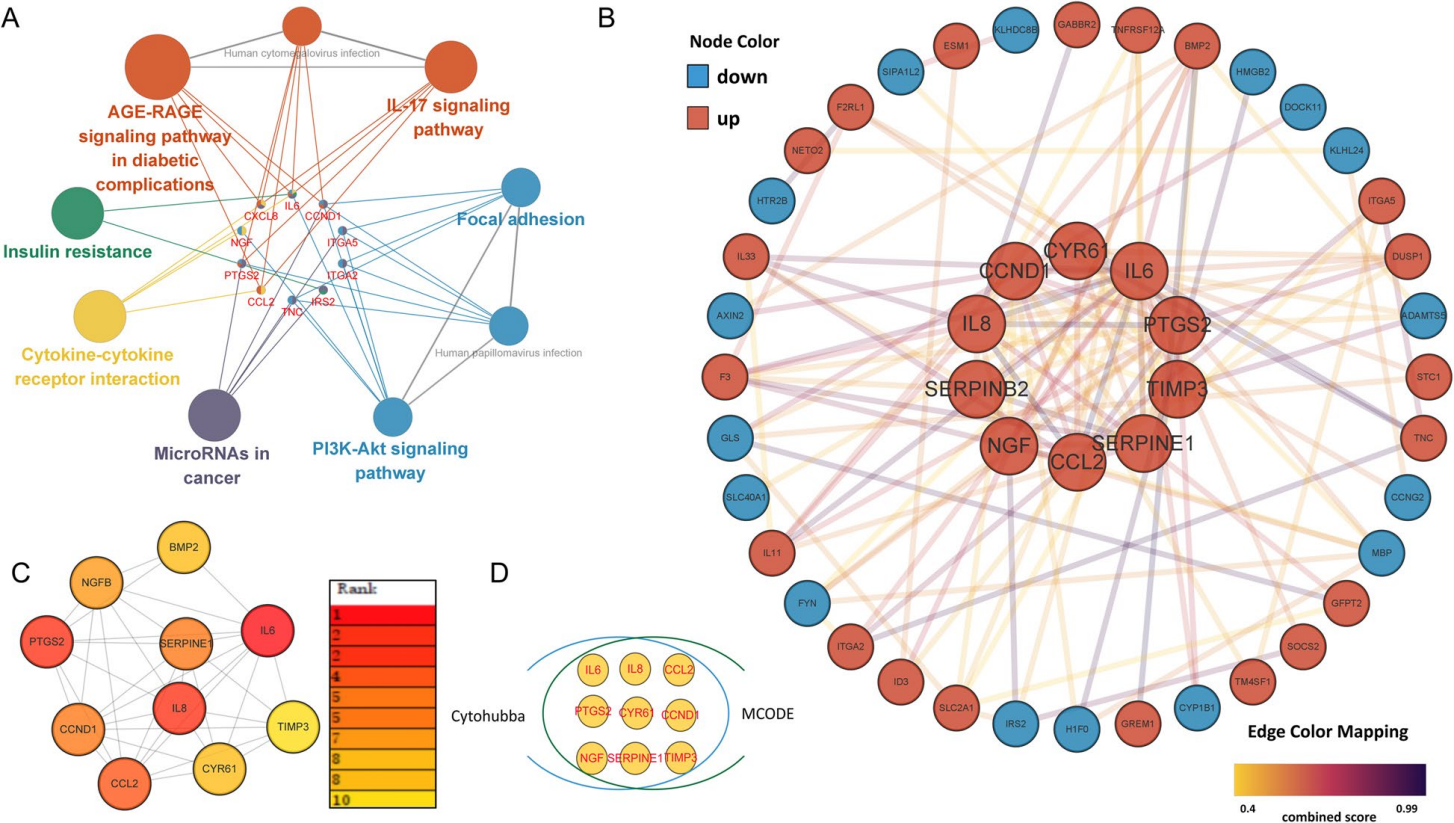
**A** The network generated by ClueGo and Cluepedia shows the pathways of KEGG enrichment and the genes shared between them. **B** The PPI network of DEGs, which was based on String, consisted of 46 nodes and 123 edges. Different node colors represent up-regulated and down-regulated DEGs, while the edge color mapping represents the combined score. A sub-cluster of 10 genes, which is shown in the center of the network, is detected by MCODE(MCODE score ≥ 8). **C** Cytohubba identified the top 10 genes in the PPI network, ranked by node degree. **D** Based on the intersection of Cytohubba and MCODE, we identified 9 up-regulated genes as hub genes

**Fig. 6:**
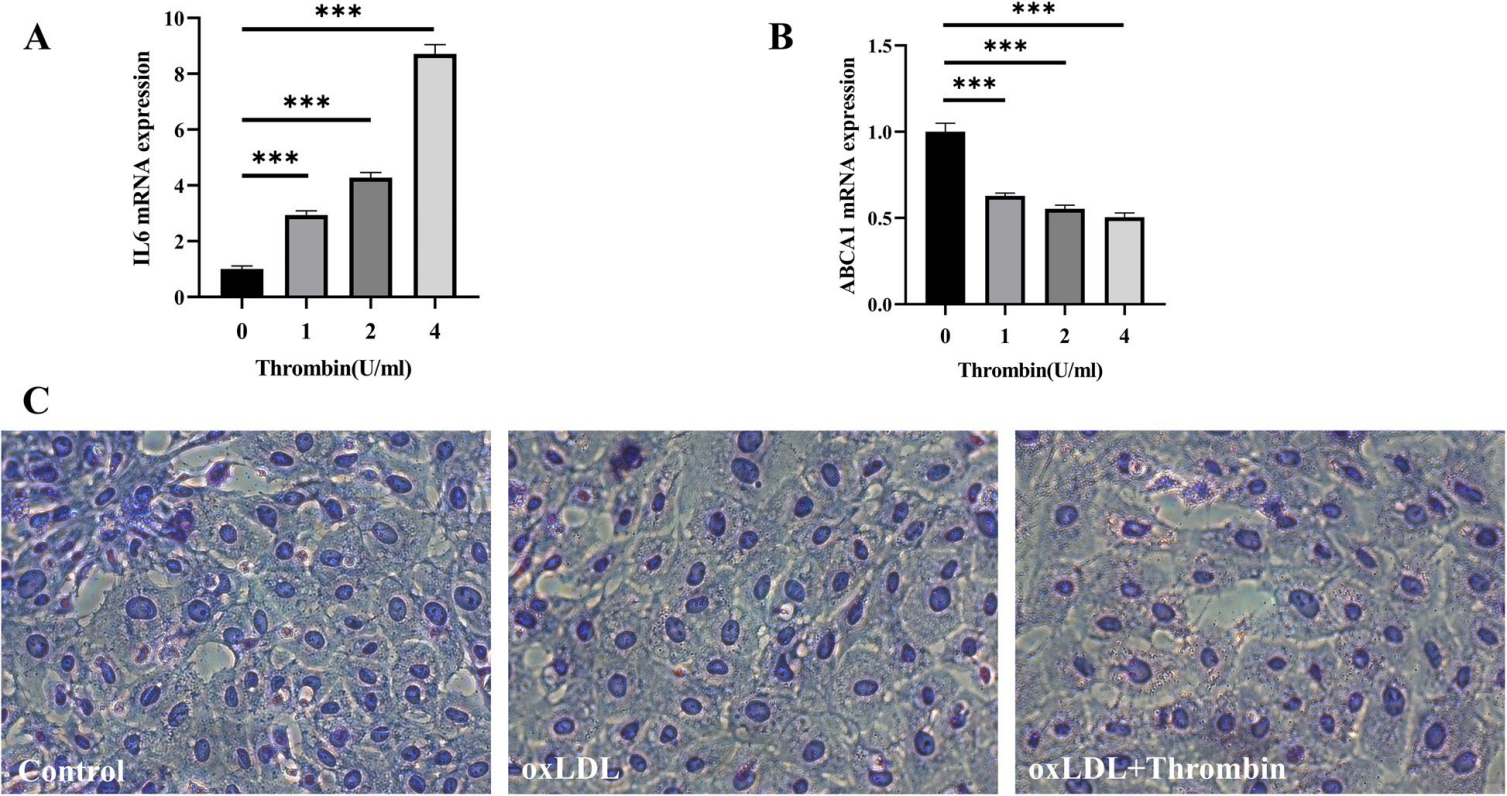
The expression of IL-6 (**A**) and ABCA1 (**B**) mRNA in RA-VSMCs after exposure to 2U/ml thrombin was determined by RT-PCR * *p* < 0.05, ** *p* < 0.01, and ***p < 0.005. **C** RA-VSMCs were stimulated with ox-LDL in the presence or absence of thrombin(2U/ml). Red stain of Oil Red O denotes the foam cell positive cells. Magnification × 40

Thrombin induced an inflammatory response in HASMCs according to the enrichment analysis results. The up-regulated genes were enriched to the GO terms such as regulation of cell adhesion (GO:0030155)and regulation of leukocyte migration (GO:0002685) (Fig. 2A). In addition to affecting the hemostasis and coagulation process, GSEA results suggested that thrombin promoted the activation trend of biological process GO terms such as monocyte chemotaxis (GO:0002548), mononuclear cell migration (GO:0071674), positive regulation of acute inflammatory response(GO:0002675), positive regulation of leukocyte chemotaxis (GO:0002690), and leukocyte migration (GO:0050900) (Fig. 3A). Previous studies have shown that thrombin mediates the expression of Interleukin (IL) IL-6, CCL2/MCP-1, CXCL8/IL8, and IL33 in HASMCs via PI3K-Akt, MAPK, and other signaling pathways [1, 25, 26]. In our study, the genes of these cytokines are all significantly up-regulated in the thrombin group, especially IL6 (Fig. 4A1 and A2). At the same time, KEGG enrichment analysis and GSEA results showed that multiple signaling pathways such as PI3K-Akt, IL17, TNF, NF-KappaB, and MAPK were activated (Fig. 3B). Moreover, we found that the expression of IL11 and PTGS2 genes were also significantly up-regulated  (Fig. 4A1 and A2). IL6, IL8/CXCL8, CCl2/MCP-1, and PTGS2 were included in the hub genes identified by PPI analysis (Fig. 5D). To further validate the proinflammatory roles of thrombin in the VSMCs, IL6 mRNA was examined by RT-qPCR.After exposure to thrombin (1, 2 or 4 U/ml), levels of IL-6 mRNA (Fig. 6A) in RA-VSMCs were increased in a dose-dependent manner. These results indicate that thrombin induces HASMCs to synthesize pro-inflammatory cytokines and chemokines, which induces leukocyte migration and adhesion to inflammatory sites such as atherosclerotic plaques.

### Thrombin promotes cell proliferation and angiogenesis by up-regulating the expression of cyclin D1 and CCN1

According to GSEA results, G1/S transition of mitotic cell cycle (GO:0000082), positive regulation of DNA biosynthesis (GO:2000573), and DNA replication (hsa03030) were activated (Fig. 3). Multiple studies have shown that thrombin can promote the proliferation and migration of HASMCs via NF-kappa B, PI3K-Akt, and MAPK signaling pathways [1, 6, 7], consistent with our GSEA results (Fig. 3B). Recent studies have shown that LMCD1, one of our study’s top 5 up-regulated genes (Fig. 1D), is required for thrombin-induced smooth muscle cell proliferation [27, 28]. Additionally, GESA showed that the VEGF (vascular endothelial growth factor) signaling pathway was activated (Fig. 3B), suggesting that thrombin was involved in angiogenesis. Previous studies have shown that thrombin promotes angiogenesis both in vitro and in vivo [1]. Among the hub genes in the PPI network, except genes that encode cytokines, some genes are related to cell proliferation and angiogenesis, such as CCND1 and CCN1/CYR61 (Fig. 5D). The protein encoded by CCND1 is cyclin D1, which belongs to the highly conserved cyclin family [29]. The communication network factor 1(CCN1), also known as CYR61 (cysteine-rich protein 61), is the first identified member of the CCN family and the first member described to be an angiogenic factor [30]. It indicates that Cyclin D1 and CCN1 may play a key role in thrombin-induced smooth muscle cell proliferation and angiogenesis.

### Thrombin promotes foam cell formation by inhibiting lipid hydrolysis and autophagy

ATP binding cassette transporter (ABCA1) plays a vital role in cellular cholesterol homeostasis by transferring phospholipids and cholesterol from cell membranes to apolipoprotein A-I and high-density lipoprotein (HDL) particles [31]. Transcription factors control the expression of the ABCA1 gene, the most important of which is liver X-receptor (LXR), including LXRα and LXRβ (NR1H3 and NR1H2, respectively) [32]. In addition to the intracellular accumulation of cholesterol ester caused by ABCA1 and LXRs deficiency, mechanisms of foam cell formation include the excessive influx of modified low-density lipoprotein (LDL) due to the overexpression of scavenging receptors (SRs), such as SR-A1, CD36, and LOX1 [33]. According to our results, ABCA1 and LXRα genes were slightly down-regulated by thrombin. In contrast, LXRβ and SRs genes were not affected as a result (Fig. 4B1 and B2). To further validate our results, ABCA1 mRNA was examined by RT-qPCR.After exposure to thrombin (1, 2 or 4 U/ml), levels of ABCA1 mRNA (Fig. 6B) in RA-VSMCs were suppressed in a dose-dependent manner.To verify the effect of thrombin on foam cell formation, the RA-VSMCs were induced by ox-LDL (50 μg/ml). The oil red O-positive droplets in cells were increased after ox-LDL induction for 24 h. As shown by light microscopic photographs, the red stain droplets were more prominent in oxLDL groups, whereas thrombin treatment (2U/ml) significantly increased positive staining compared with ox-LDL group (Fig. 6C).

A recent report suggests that inhibiting the use of fatty acids as substrates by macrophages may be another mechanism by which thrombin induces smooth muscle-derived foam cell formation [34]. In our study, the down-regulated genes of DEGs were enriched to the GO terms such as positive regulation of hydrolase activity (GO:0051345)and regulation of lipid metabolic process (GO:0019216) (Fig. 2C). The GSEA indicated lipid catabolic process (GO:0016042), fatty acid catabolic process (GO:0009062), lipid oxidation (GO:0034440), fatty acid oxidation (GO:0019395) and Fatty acid degradation (hsa00071) were suppressed (Fig. 3).

Excess intracellular cholesterol will be esterified and stored in lipid droplets (LDs) as non-cytotoxic cholesterol esters (CEs). Enhancing lipid droplet-associated cholesteryl ester hydrolysis increases cholesterol efflux. Neutral hydrolases are responsible for the hydrolysis of cholesterol esters in the cytoplasm, while autophagy, induced explicitly by lipid overload, can mediate the lysosomal hydrolysis of cholesterol esters [35]. The autophagy activators High Mobility Group Box 1(HMGB1) and High Mobility Group Box 2(HMGB2) gene knockout promoted foam cell formation, and SERPINH1 (Serpin Family H Member 1) was specifically enriched on LDs following autophagy inhibition [36]. In our study, HMGB2 gene expression was significantly down-regulated by the thrombin (Fig. 4C1 and C2). In addition, we examined the gene expression of SQSTM1/P62 (sequestosome1) and MAP1LC3A/LC3(microtubule-associated protein 1 light chain 3), which are commonly used as autophagosome markers. The results showed that thrombin slightly down-regulated the gene expression of P62 (Fig. 4C1 and C2). Terms such as autophagy (GO:0006914) process utilizing autophagic mechanism (GO:0061919), positive regulation of autophagy (GO:0010508), and autophagosome assembly (GO:0000045) were suppressed in GSEA (Fig. 3A). These results suggest that thrombin may reduce cholesterol efflux by affecting lipid hydrolysis and autophagy, thereby promoting foam cell formation.

## Discussion

AS is the common pathological basis for the occurrence and development of cardiovascular and cerebrovascular diseases. Stroke and coronary heart disease caused by AS have become the leading cause of death and disability in humans [37]. Atherosclerosis is accompanied by a chronic, low-grade inflammatory response that attracts cells from innate and acquired immune systems into atherosclerotic plaques, which exacerbates atherosclerotic progression [4]. SMCs, which secrete various cytokines and chemokines under the stimulation of pro-inflammatory factors, play an essential role in the inflammatory response of atherosclerosis [38]. Thrombin, a trigger of vascular wall inflammation, has been shown to increase the expression of IL6, CCL2/MCP-1, CXCL8/IL8, and IL33 in HASMCs [1, 25, 26].

IL-6 drives SMC into a proinflammatory and proliferative state by trans-signaling pathway [39]. Although the mechanism by which IL6 affects AS is unclear, inhibition of IL­6 trans­signalling reduces the incidence of AS, indicating that IL­6 trans­signalling might have a pathogenic role in atherosclerosis [40, 41]. According to concordant Mendelian randomization studies, IL-6 participates in human cardiovascular events causally. The increased risk of cardiovascular disease associated with the presence of clonal hematopoiesis of indeterminate potential, a potent, common, age-related, independent, and newly recognized risk factor, is abrogated in patients with a loss of function mutation in IL6 [40]. Patients with atherosclerosis show a higher serum level of IL8, which induces the formation of neutrophil extracellular traps that aggravate atherosclerosis progression in vivo [42]. According to an in vitro study, IL8 may promote the formation of foam cells by inhibiting the cholesterol efflux protein ABCA1 [43]. IL-33, a member of the recently discovered IL-1 cytokine family, is highly expressed in human atherosclerotic plaques.IL-33 is involved in the pathophysiology of vascular diseases through endothelial dysfunction and VSMC migration [26]. CCL2, also known as the monocyte chemoattractant protein-1 (MCP-1), is associated with an increased risk of cardiovascular events and atherosclerotic plaque instability in humans [38]. CCl2 has been demonstrated to influence atherosclerosis progression through its effects on monocyte trafficking and lipid deposition in atherosclerotic plaques using different mouse models [44].

**Fig. 7:**
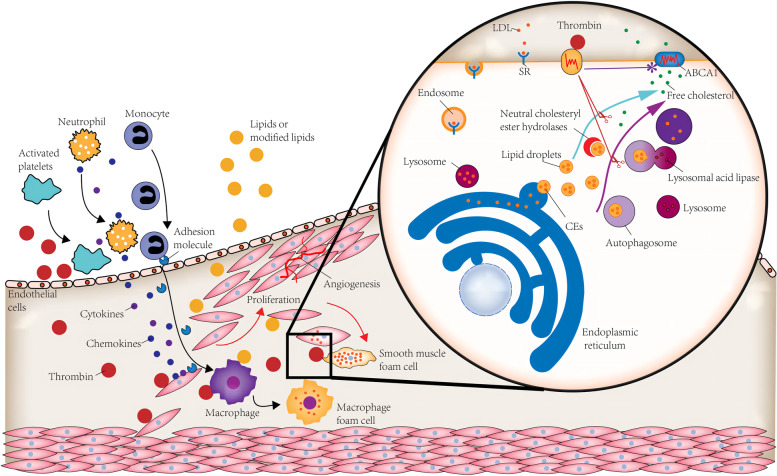
Effect of thrombin on smooth muscle cells(SMCs) in the pathogenesis of atherosclerosis. Thrombin induces the synthesis of pro-inflammatory cytokines and chemokines in SMCs, including IL6, IL8/CXCL8, IL33, CCl2/ MCP-1, IL11, and PTGS2, which in turn induces monocytes and neutrophil migration and adhesion to inflammatory sites. Thrombin promotes SMCs proliferation and angiogenesis in atherosclerotic plaque. LDL intake by scavenger receptor (SR) is delivered to the lysosome (LY), where it is hydrolyzed to free cholesterol before entering the endoplasmic reticulum(ER). Subsequently, lipid droplets(LDs) rich in cholesterol esters(CEs) are formed from the ER. There are two pathways of LD lipolysis: 1. Lipase-mediated intracytoplasmic hydrolysis of LD-associated neutral lipids; 2. Autophagosome mediates the cytoplasmic transport of LD to the lysosome, where LD cholesterol ester is hydrolyzed by lysosomal acid lipase. Thrombin may inhibit both pathways. Eventually, free cholesterol is excreted from the cell by ABCA1

We have some new findings on thrombin promoting the proinflammatory phenotype of HASMCs. We observed that thrombin-stimulated HASMC overexpressed not only IL6, CCL2/MCP-1, CXCL8/IL8, and IL33 but also IL11 and PTGS2 (Fig. 4A1 and A2). PTGS2 is one of the hub genes identified by PPI analysis (Fig. 5D). Prostaglandin-endoperoxide synthase 2(PTGS2), also known as cyclooxygenase2 (COX2), is the crucial enzyme in prostaglandin biosynthesis and acts as a dioxygenase and as a peroxidase [29]. Prostaglandins (PGS), especially Prostaglandin E2(PGE2), play a critical role in the inflammatory response and regulate cardiovascular function [45]. PTGS2, which converts arachidonate to prostaglandin H2 (PGH2), is typically overexpressed in atherosclerotic lesions [46]. PGH2 is converted to PGE2 by PGE synthase, producing proinflammatory and anti-inflammatory effects. PGE2 induces the expression of matrix metalloproteinases, which are crucial in degrading plaque stability. In addition, PGE2 promotes angiogenesis by inducing angiogenic factors [46]. IL-11 is a member of the glycoprotein (GP) 130 cytokine family, which also includes IL-6 [47]. IL11 was previously thought to have an anti-inflammatory effect on the cardiovascular system, but studies have shown that when it is expressed in VSMC, it induces a proinflammatory response. Additionally, IL11 is required for VSMC to lose its contractile properties and differentiate into a synthetic phenotype mainly characterized by secretion of extracellular matrix, increased proliferation, and migration. In AS, this cellular transition plays a vital role in the pathophysiology of adverse aortic remodeling [48]. In summary, thrombin converts HSMCs from contractile phenotype to synthetic phenotype, which enables HSMCs to synthesize IL6, IL8, CCL2, and IL33 and may lead to the release of IL11 and PGTS2. This conversion eventually leads to the progression of as and the instability of atherosclerotic plaque (Fig. 7).

It is well known that thrombin promotes the proliferation and migration of VSMCs in atherosclerotic plaques [1, 6]. We found for the first time that CCND1 and CCN1/CYR61 play a crucial role in thrombin-induced smooth muscle cell proliferation and migration in our study. These two genes are contained in the hub gene identified by PPI analysis (Fig. 5D). The protein encoded by CCND1 is cyclin D1 [29], which forms a complex with the regulatory subunits of cyclin-dependent kinase (CDK)4 or CDK6, leading to the phosphorylation of Rb protein and activation of the E2F transcription factor family, thereby promoting the cell to enter the S phase of the cell cycle [49]. Thus, increased expression of cyclin D1 can enhance cell cycle progression and cell proliferation. Neoangiogenesis is closely associated with plaque progression. The incomplete maturation and the fragility of neo capillaries promote intraplaque hemorrhages, leading to plaque instability and rupture [50]. CCN1 is essential in cell proliferation, adhesion, inducing angiogenesis, and other critical physiological activities [51]. The Serum level of CCN1 in rheumatoid arthritis patients was positively correlated with carotid intima-media thickness (CIMT) [52]. In atherosclerotic plaque, thrombin promotes proliferation and angiogenesis of HASMC, eventually leading to plaque instability (Fig. 7).

**Fig. 8:**
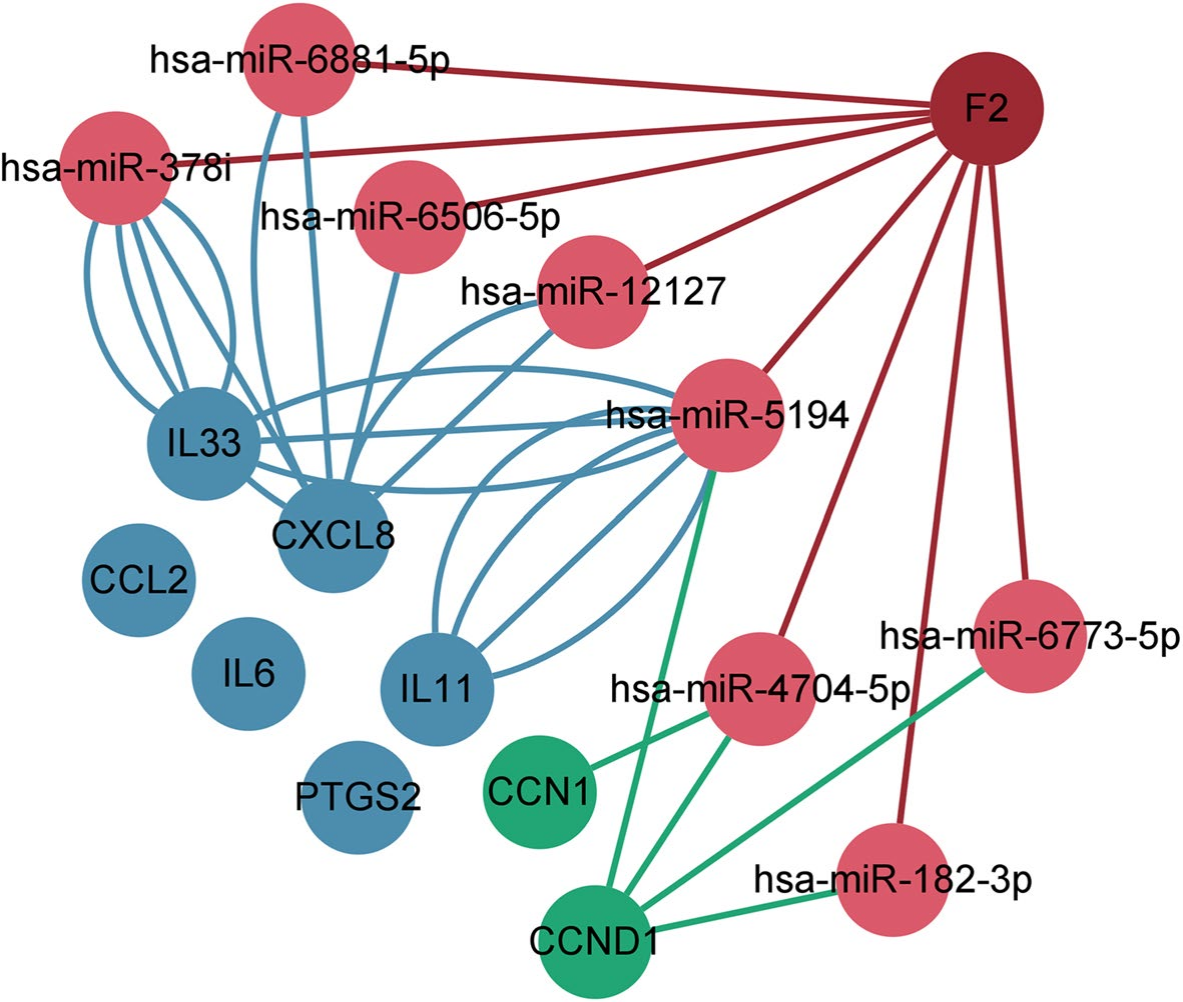
The miRNA regulatory network between thrombin (F2) and the target genes

Micro-ribonucleic acids(miRNAs) are recognized as post-transcriptional gene expression regulators. Many miRNAs have emerged as potential therapeutic targets and new biomarkers for heart and vascular disease [53]. Some miRNAs have been shown to play a role in thrombin-related pathophysiological processes.Mirna-146, for instance, plays a vital role in the regulation of thrombin-induced endothelial inflammation [54], while miRNA-181b inhibits thrombin-mediated endothelial activation and arterial thrombosis [55], and miRNA 222 is involved in thrombin regulation of the cell cycle [56]. To further investigate the regulatory role of miRNA in HASMCs after thrombin intervention, we constructed a miRNA network between thrombin and essential genes (Fig. 8). In the miRNA network, miRNA-5194 is the most critical, which participates in the mutual regulation between thrombin and pro-inflammatory cytokines and interacts with thrombin and cell cycle proteins.MiR-5194 is not only a biomarker for the diagnosis and prognosis of glioblastoma [57], but it also regulates liver lipid metabolism [58]. Together with our study, miR-5194 deserves further investigation and might become a potential therapeutic target for AS.

The most important finding of our research is that thrombin may reduce cholesterol efflux by affecting lipid hydrolysis and autophagy, thereby promoting foam cell formation. Foam cells play an essential role in all stages of the development of AS lesions. Although many cell types can be transformed into foam cells from initial lesions to late plaques, foam cells derived from monocytes/macrophages have always been considered the main factor in the occurrence and development of AS plaques. However, many recent studies have shown that the proportion of foam cells derived from VSMCs is more than 50% in AS plaques, which plays a more critical role, but its formation mechanism is not fully understood [59, 60]. The formation of foam cells is mainly caused by the excessive influx of modified low-density lipoprotein into cells and the accumulation of intracellular cholesterol esters. VSMCs express SRs and thus can take up intimal lipoproteins like macrophages [61]. Other mechanisms, including microphagocytosis of serum lipoproteins and aggregation of low-density lipoproteins via LDL receptor associated protein-1 uptake [62], are involved in smooth muscle cell-derived foam cell formation.ABCA1 plays a crucial role in cholesterol extravasation, resulting in an increased propensity to transform into foam cells. A synthetic phenotype of VSMC metabolizes lipids differently, partly due to reduced expression of cholesterol esterase and reduced levels of ABCA1 [60]. Raghavan et al. performed in vitro and in vivo experiments, demonstrating for the first time that thrombin can induce the formation of smooth muscle-derived foam cells [8]. Boro et al. found that thrombin-induced ABCA1 phosphorylation led to its ubiquitination and degradation [9]. Our study showed that in addition to promoting the degradation of ABCA1, thrombin also decreased the transcription of ABCA1 and LXRα but had no significant effect on the expression of scavenger receptors (Fig. 4B1 and B2).

Excessive intake of LDL by scavenger receptors results in the imbalance of the compensatory metabolic mechanism of cells [61]. LDL is transported to the lysosome (LY), where LDL cholesterol esters are hydrolyzed to release free cholesterol. Free cholesterol then exits LY and moves to the endoplasmic reticulum (ER). Subsequently, LDs rich in non-toxic cholesterol esters are formed from the ER.LDs lipolysis is a critical step in regulating foam cell formation [35]. Additionally to neutral lipase mediating the hydrolysis of neutral lipids associated with LDs, autophagy is also involved in transporting cytoplasmic LDs to the lysosome [36] (Fig. 7). Some studies indicate that thrombin can induce autophagy in astrocytes and increase the conversion of LC3I to LC3II in hypoxia/reoxygenation-injured cardiomyocytes [63, 64]. However, limited research has addressed the regulatory roles of thrombin and autophagy in VSMCs.Our study’s GSEA and KEGG results indicate that thrombin activates the PI3K-Akt signaling pathway and inhibits autophagy in SMCs (Figs. 3 and 5A). Therefore, we thought it would be fascinating to investigate how thrombin affects autophagy in VSMCs.According to a recent study, the P2RY12 receptor promotes VSMC-derived foam cell formation and lipid accumulation by inhibiting autophagy in advanced atherosclerosis through the PI3K-Akt-MTOR signaling pathway [65]. Based on our enrichment analysis, we speculated that thrombin might promote the formation of smooth muscle-derived foam cells by inhibiting lipid hydrolysis and autophagy via the PI3K-Akt signaling pathway. Whatever the case may be, thrombin does play an essential role in lipid metabolism.

Researchers are exploring the role of thrombin antagonists in the progression of atherosclerosis. There are many thrombin antagonists, among which dabigatran is one of the most studied recently. In vivo experiments confirmed that dabigatran attenuates atherosclerosis in ApoE deficiency mice [66] and protects against high-fat diet-induced fatty liver disease in mice [67]. Clinical evidence has shown that dabigatran reduces serum ApoB levels [68]. However, like other thrombin antagonists, dabigatran is at risk of bleeding [69]. Recent research has also shown direct thrombin inhibitors, including bivalirudin, ximelagatran, and dabigatran, increase the risk of myocardial infarction [70]. Although this is controversial, direct thrombin inhibitors have many shortcomings and can not be safely used to prevent atherosclerosis. In our opinion, even though thrombin can promote atherosclerosis, its primary role remains procoagulant and anticoagulant. So the major indication for this drug is stroke prevention in arterial fibrillation. Abnormal proliferation of SMCs contributes to the progression of atherosclerotic plaques and narrowing of the arterial lumen. SMC proliferation, however, may also be beneficial to advancing AS, such as preventing the rupture of the fibrous cap [5]. Therefore, we believe that thrombin inhibitor not only inhibits the proliferation of SMCs, but also destroys the stability of atherosclerotic plaques, leading to the rupture and bleeding of plaques, and ultimately increasing the incidence of myocardial infarction. Based on the above reasons, we believe that direct thrombin inhibitors cannot be used to prevent atherosclerosis. The development of mild, indirect thrombin inhibitors may be a good direction for anti-atherosclerosis. Baicalin, which is a natural bioactive compound of S. baicalensis Georgi (SBG), exerted a protective effect against thrombin-induced VSMC inflammation as resulting from the upregulation of PAR-1 [71].

## Conclusions

One of the limitations of our study is that the data used in our analysis are obtained from a public database, and the sample size is small. Meanwhile, our results cannot be verified due to the lack of experiments. We conclude that thrombin promotes HASMC transformation into a synthetic phenotype. As a result, HASMCs produce pro-inflammatory mediators, promoting leukocyte migration and adhesion to atherosclerotic plaques. Moreover, our results suggest that thrombin promotes HASMC proliferation and angiogenesis in atherosclerotic plaques.

Furthermore, we infer that thrombin has a significant effect on lipid metabolism. Aside from promoting ABCA1 hydrolysis, thrombin may inhibit lipid hydrolysis and autophagy, leading to smooth muscle-derived foam cell formation (Fig. 7). In conclusion, the effect of thrombin on SMC is complex and variable. As a therapeutic target of atherosclerosis, thrombin’s role in the pathogenesis of atherosclerosis is worthy of further study.

## Data Availability

The datasets used or analysed during the current study are available from the corresponding author on reasonable request.

## Abbreviations

HASMCs: Human aortic smooth muscle cells
AS: Atherosclerosis
DEGs: Differentially expressed genes
PARs: Protease-activated receptors
SMCs: Smooth muscle cells
GEO: Gene Expression Omnibus
GO: Gene Ontology
KEGG: Kyoto Encyclopedia of Genes and Genomes
GSEA: Gene set Enrichment Analysis
PPI: Protein–protein interaction
PCA: Principal component analysis
FC: Fold change
RA-VSMCs: Rat aorta vascular smooth muscle cells
VEGF: Vascular endothelial growth factor
CCN1: Communication network factor 1
CYR61: Cysteine-rich protein 61
ABCA1: ATP binding cassette transporter
HDL: High-density lipoprotein
LXR: Liver X-receptor
LDL: Low-density lipoprotein
SRs: Scavenging receptors
LDs: Lipid droplets
CEs: Cholesterol esters
HMGB1: High Mobility Group Box 1
HMGB2: High Mobility Group Box 2
SERPINH1: Serpin Family H Member 1
SQSTM1: Sequestosome1
MAP1LC3A: Microtubule-associated protein 1 light chain 3
MCP-1: Monocyte chemoattractant protein-1
PTGS2: Prostaglandin-endoperoxide synthase 2
COX2: Cyclooxygenase2
PGS: Prostaglandins
PGE2: Prostaglandin E2
PGH2: Prostaglandin H2
GP: Glycoprotein
CDK: Cyclin-dependent kinase
CIMT: Carotid intima-media thickness
miRNAs: Micro-ribonucleic acids
LY: Lysosome
ER: Endoplasmic reticulum
